# Supplementary material for: Trustworthy and ethical AI-enabled cardiovascular care: a rapid review
Source: BMC Med Inform Decis Mak. 2024 Sep 4;24:247. doi: 10.1186/s12911-024-02653-6 (PMC11373417; doi:10.1186/s12911-024-02653-6)
Supplement: Supplementary file 1 — Supplementary Material 1. [file 12911_2024_2653_MOESM1_ESM.docx]

**Trustworthy and Ethical AI-enabled Cardiovascular Care: A Rapid Review**

Maryam Mooghali, MD, MSc*^1^; Austin M. Stroud, MA^2^; Dong Whi Yoo, PhD, MS, MFA^3^; Barbara A Barry, PhD^4,5^; Alyssa A. Grimshaw, MSLIS^6^; Joseph S. Ross, MD, MHS^1,7^; Xuan Zhu, PhD, MS^4^; Jennifer E. Miller, PhD^1^

^1^ Section of General Internal Medicine, Department of Internal Medicine, Yale School of Medicine, New Haven, CT, United States

^2^ Biomedical Ethics Research Program, Mayo Clinic, Rochester, MN, United States

^3^ School of Information, Kent State University, Kent, OH, United States

^4^ Robert D. and Patricia E. Kern Center for the Science of Health Care Delivery, Mayo Clinic, Rochester, MN, United States

^5^ Division of Health Care Delivery Research, Mayo Clinic, Rochester, MN, United States

^6^ Harvey Cushing/John Hay Whitney Medical Library, Yale University, New Haven, CT, United States

^7^ Department of Health Policy and Management, Yale School of Public Health; and Center for Outcomes Research and Evaluation, Yale-New Haven Health System, New Haven, CT, United States

***Corresponding author:** Maryam Mooghali, MD, MSc; Yale Center for Outcomes Research and
Evaluation (CORE), 195 Church Street, New Haven, CT, United States. Zip code: 06510; Phone: (203) 497-1239; email: maryam.mooghali@yale.edu.

**Appendix 1**

**Ovid Embase**

1 (exp cardiovascular disease/ or exp cardiac equipment/) and (exp artificial intelligence/ or exp machine learning/)

2 (heart or cardiac or myocard* or arrhythmia* or valvular or coronar* or cardio* or ventric* or hypertens* or vascular or angiogra* or echocardiogra* or electrocardiogra* or EKG or ECG or pacemaker*).tw,kf.

3 ((artificial or computat* or computer* or machine or deep or transfer or hierarchical) adj3 (intelligence* or learning* or reasoning* or diagnosis or diagnoses or simulation* or interface* or assist*)).tw,kf.

4 (neural network* or random forest* or decision tree* or knowledge representation* or computer vision system* or computer reasoning*).tw,kf.

5 ((predict* or prognost* or clinical decision) adj3 (support* or system* or model* or tool* or scoring or calculation* or algorithm*)).tw,kf.

6 AI.ti,ab.

7 3 or 4 or 5 or 6

8 2 and 7

9 1 or 8

10 exp patient attitude/

11 (exp trust/ or exp knowledge/ or exp perception/ or public opinion/ or uncertainty/) and exp patient/ 40190

12 ((patient* or consumer* or care-giver* or caregiver*) adj3 (accept* or adherence or attitude* or awareness* or barrier* or behavior* or behaviour* or belief* or choice* or compliance or confidence or decision* or dislik* or distrust* or doubt* or facilitator* or fear* or hesita* or knowledge or mistrust* or motivat* or nonadherence or non-adherence or noncompliance or non-compliance or opinion* or perception* or preference* or reluctan* or refus* or trust* or understand* or understood or uptak* or willingness)).tw,kf.

13 10 or 11 or 12

14 9 and 13

**Ovid MEDLINE(R) ALL**

1 exp cardiovascular diseases/ and (exp artificial intelligence/ or exp machine learning/)

2 (heart or cardiac or myocard* or arrhythmia* or valvular or coronar* or cardio* or ventric* or hypertens* or vascular or angiograph*).tw,kf.

3 ((artificial or computat* or computer* or machine or deep or transfer or hierarchical) adj3 (intelligence* or learning* or reasoning* or diagnosis or diagnoses or simulation* or interface* or assist*)).tw,kf.

4 (neural network* or random forest* or decision tree* or knowledge representation* or computer vision system* or computer reasoning*).tw,kf.

5 ((predict* or prognost* or clinical decision) adj3 (support* or system* or model* or tool* or scoring or calculation* or algorithm*)).tw,kf.

6 3 or 4 or 5

7 2 and 6

8 1 or 7

9 exp Health Knowledge, Attitudes, Practice/

10 exp Attitude to Health/

11 (exp trust/ or exp knowledge/ or exp perception/ or public opinion/ or uncertainty/) and exp patient/

12 ((patient or consumer or care-giver* or caregiver*) adj3 (accept* or adherence or attitude* or awareness* or barrier* or behavior* or behaviour* or belief* or choice* or compliance or confidence or decision* or dislik* or distrust* or doubt* or facilitator* or fear* or hesita* or knowledge or mistrust* or motivat* or nonadherence or non-adherence or noncompliance or non-compliance or opinion* or perception* or preference* or reluctan* or refus* or trust* or understand* or understood or uptak* or willingness)).tw,kf.

13 or/9-12

14 8 and 13

**Scopus**

( TITLE-ABS-KEY ( ( patient* OR consumer* OR care-giver* OR caregiver* ) W/3 ( accept* OR adherence OR attitude* OR awareness* OR barrier* OR behavior* OR behaviour* OR belief* OR choice* OR compliance OR confidence OR decision* OR dislik* OR distrust* OR doubt* OR facilitator* OR fear* OR hesita* OR knowledge OR mistrust* OR motivat* OR nonadherence OR non-adherence OR noncompliance OR non-compliance OR opinion* OR perception* OR preference* OR reluctan* OR refus* OR trust* OR understand* OR understood OR uptak* OR willingness ) ) ) AND ( TITLE-ABS-KEY ( ( artificial OR computat* OR computer* OR machine OR deep OR transfer OR hierarchical ) W/3 ( intelligence* OR learning* OR reasoning* OR diagnosis OR diagnoses OR simulation* OR interface* OR assist* ) ) OR TITLE-ABS-KEY ( "neural network*" OR "random forest*" OR "decision tree*" OR "knowledge representation*" OR "computer vision system*" OR "computer reasoning*" ) ) AND ( TITLE-ABS-KEY ( heart OR cardiac OR myocard* OR arrhythmia* OR valvular OR coronar* OR cardio* OR ventric* OR hypertens* OR vascular OR angiogra* OR echocardiogra* OR electrocardiogra* OR ekg OR ecg OR pacemaker* ) )

**Web of Science Core Collection**#1 TS=(heart or cardiac or myocard* or arrhythmia* or valvular or coronar* or cardio* or ventric* or hypertens* or vascular or angiogra* or echocardiogra* or electrocardiogra* or EKG or ECG or pacemaker*)

#2 **TS=((artificial or computat* or computer* or machine or deep or transfer or hierarchical) near/3 (intelligence* or learning* or reasoning* or diagnosis or diagnoses or simulation* or interface* or assist*)) or TS=("neural network*" or "random forest*" or "decision tree*" or "knowledge representation*" or "computer vision system*" or "computer reasoning*") or TS=**

**((predict* or prognost* or "clinical decision") near/3 (support* or system* or model* or tool* or scoring or calculation* or algorithm*))**

**#3 TS=((patient* or consumer* or care-giver* or caregiver*) near/3 (accept* or adherence or attitude* or awareness* or barrier* or behavior* or behaviour* or belief* or choice* or compliance or confidence or decision* or dislik* or distrust* or doubt* or facilitator* or fear* or hesita* or knowledge or mistrust* or motivat* or nonadherence or non-adherence or noncompliance or non-compliance or opinion* or perception* or preference* or reluctan* or refus* or trust* or understand* or understood or uptak* or willingness))**

**#4 #1 and #2 and #3**

**Cochrane Library**

#1 ((patient* or consumer* or care-giver* or caregiver*) near/3 (accept* or adherence or attitude* or awareness* or barrier* or behavior* or behaviour* or belief* or choice* or compliance or confidence or decision* or dislik* or distrust* or doubt* or facilitator* or fear* or hesita* or knowledge or mistrust* or motivat* or nonadherence or non-adherence or noncompliance or non-compliance or opinion* or perception* or preference* or reluctan* or refus* or trust* or understand* or understood or uptak* or willingness)):ti,ab

#2 ((artificial or computat* or computer* or machine or deep or transfer or hierarchical) near/3 (intelligence* or learning* or reasoning* or diagnosis or diagnoses or simulation* or interface* or assist*)):ti,ab or ("neural network*" or "random forest*" or "decision tree*" or "knowledge representation*" or "computer vision system*" or "computer reasoning*"):ti,ab or

((predict* or prognost* or "clinical decision") near/3 (support* or system* or model* or tool* or scoring or calculation* or algorithm*)):ti,ab

#3 (heart or cardiac or myocard* or arrhythmia* or valvular or coronar* or cardio* or ventric* or hypertens* or vascular or angiogra* or echocardiogra* or electrocardiogra* or EKG or ECG or pacemaker*):ti,ab

#4 #1 and #2 and #3

**Google Scholar**

Artificial intelligence cardiovascular trust
